# Controlled Silver Nanoparticle Formation in Hair Fibers Dyed with *Reseda luteola* L.: A Study on Additive-Dependent Penetration and Aggregation

**DOI:** 10.3390/molecules30163446

**Published:** 2025-08-21

**Authors:** Julia Katharina Hachmann, Charlotte Ruhmlieb, Volkmar Vill, Fabian Straske

**Affiliations:** 1 Department of Chemistry, University of Hamburg, Martin-Luther-King-Platz 6, 20146 Hamburg, Germany; julia.hachmann@uni-hamburg.de (J.K.H.); charlotte.ruhmlieb@uni-hamburg.de (C.R.); volkmar.vill@uni-hamburg.de (V.V.); 2 Henkel AG & Co KGaA, Ruhrstraße 19, 22761 Hamburg, Germany

**Keywords:** nanoparticles, *Reseda luteola* L., TEM-imaging, microtome cuts, silver, hair dye

## Abstract

Applying physico-analytical methods to whole hair fibers enables investigation of hair dye performance. Light microscopy, SEM imaging and EDX mapping of intact hair fibers, as well as TEM imaging of microtome cuts, provided insights into the distribution, size, shape and growth patterns of the dyeing species and particles, thus demonstrating the correlation between silver nanoparticles (AgNPs) and dye impression. Yak hair fibers were treated with a polyphenol-containing *Reseda luteola* L. extract (RE), which had been acidified using either hydrochloric acid (HCl) or citric acid (CA), and subsequently exposed to silver nitrate (AgNO_3_), resulting in the formation of quasi-spherical silver nanoparticles (AgNPs) that were depicted several microns deep inside the hair fiber, regardless of the additive used. The particles appeared to aggregate preferentially in sulfur-rich domains within the hair fiber, probably due to the affinity of silver ions on the NP’s surface towards sulfur. The additives significantly affected the size and aggregation behavior of the particles. Using HCl, larger, aggregated particles were formed, whereas the application of CA yielded smaller, more uniform particles and a higher penetration depth. Despite different particle sizes, the dye outcome was comparable. In strands treated with HCl, washing brought the particles deeper into the hair cortex and resulted in further aggregation. Thus, HCl promoted the formation of larger particles whereas CA yielded more uniformly sized particles. These findings open a new route for metal nanoparticle-based hair dyes with excellent wash fastness.

## 1. Introduction

Although plant-based dyes have been used for thousands of years, their reaction products and mechanisms are insufficiently researched. Due to an increase in consumer-driven demand for more sustainable hair dye products [1], because of potential health [2] and environmental [3] hazards derived from oxidative dye precursors, plant-derived dyes are stepping into focus.

A special group of plant dyes is represented by mordant dyes, where a plant extract is used in combination with a metal salt to create and alter color impressions, especially on fabrics, based on the formation of complexes between phytocompounds, such as polyphenols, and the metal ion [4,5].Besides complex formation, polyphenols enable the formation of nanoparticles. As part of a redox reaction, hydroxy moieties in polyphenols are oxidized to favor the reduction in metal ions, enabling formation and growth of nanoparticles (NPs) [6]. Numerous studies have reported the use of various plant parts for nanoparticle formation, including fruits [7,8,9], roots [10,11] and leaves [12,13,14]. *Reseda luteola* L. has long been utilized as a natural dye for wool, typically together with metal salts, such as alaun, to facilitate mordanting processes [15,16,17,18]. The plant’s capacity to participate in both complex formation and nanoparticle formation has been substantiated, and is primarily attributed to its flavonoid constituents, such as luteolin and apigenin. These flavonoids have demonstrated both complexing and reductive abilities toward metal ions, thereby enabling the reactivity observed [18,19,20]. However, the application of a *Reseda luteola* L. extract in combination with silver nitrate to form silver nanoparticles on yak hair fibers for dyeing purposes has only been reported recently by our research group [21].

The shape and size of nanoparticles are deliberately controlled by the addition of capping agents that attach to the particle’s surface and provide stability. They prevent or facilitate growth and protect the particle from dissolution. Among larger molecules, such as peptides or enzymes, functional groups of molecules, such as the carboxyl groups in citrate or ions like chloride, can take on this role [6,22,23,24,25]. The major advantage of using plant material for nanoparticle synthesis lies in the fact that they can serve as both reducing agents and capping agents of the resulting nanoparticles, thus making them a favorable compound for bioinspired nanoparticle synthesis [6,26,27].

AgNPs are widely used to functionalize textiles against bacteria due to their antimicrobial activity [28,29,30,31]. Only a few studies investigate the potential of NPs as hair dyes on the whole fiber or in cross-sections [32,33], whereas more research is performed on fibers and powders as media to synthesize silver nanoparticles without dyeing intentions [34,35,36]. Hence, methods to investigate NP-based hair dyes inside the hair fiber are rare. Some methods to investigate hair fiber surface and cross-sections include, but are not limited to, SEM and EDX mapping as well as TEM, XRD and Raman spectroscopy [37,38,39,40,41,42,43,44]. The methods originate from medicinal studies to investigate the composition or anomalies of hair fibers. In particular, cuts of cross-sections of hair fibers, called microtome cuts, enable the possibility of gathering information on hair morphology and can be used to understand the penetration depth of hair dyes [45,46].

A hair fiber is mainly composed of keratinous proteins and comprises three main compartments—the cuticle, the cortex and the medulla [35]. The outermost layer, the cuticle, consists of several layers of overlapping cells, adding up to a thickness of approx. 5 µm, protecting the inlying cortex. In human hair, there are an average of six to eight individual layers, whereas in yak hair fibers that are similar to wool fibers in their morphology, less layers are depicted [44,47,48]. The cuticle itself is divided into four compartments, the epicuticle, the A-layer and the exo- and endocuticle (from outside to inside). Each layer has a different chemical composition, influencing its behavior towards external compounds. The underlying cortex comprises up to 90% of the entire hair fiber and consists of keratinous cortical cells and intercellular binding material, also called cell membrane complex (CMC), that exist between cortical cells as well as between cuticle and cortical cells [47,49]. Cortical cells contain sulfur-rich moieties, making them a favorable environment for silver. The center of the fiber marks the medulla, a cavity that is not found in every hair fiber and can have different shapes and sizes, varying from spherical to deformed [44,47]. For hair dyeing, the cuticle and cortex are the key players.

In this paper, we investigated a coloring agent in hair fiber resulting from the reaction of *Reseda luteola* L. plant extract and silver nitrate, depending on chloride and citrate as additives required for color formation. The focus lay on the application and suitability of TEM and SEM imaging as well as EDX mapping on whole hair fibers and microtome cuts to observe particles in a potentially formative environment, such as the hair fiber. Effects of the application of citrate and chloride on size and shape of the observed nanoparticles were pointed out as well as the location of particles inside the hair.

## 2. Results

### 2.1. Investigations of Hair Surface

**Figure 1:**
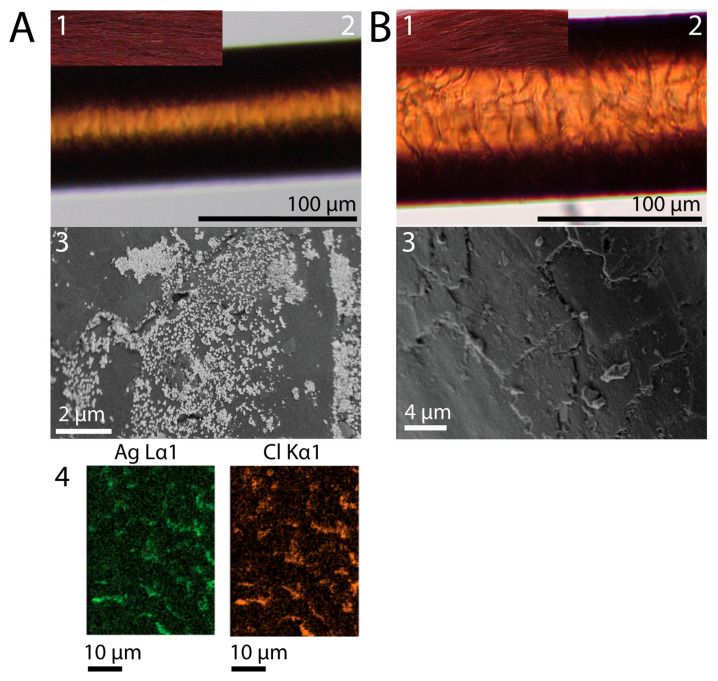
(**A**) (**1**) Photograph of dye outcome of strand treated with 1% RE at pH 3 adjusted with 10% HCl and subsequent treatment with 1% AgNO_3_, depicting a consistently red dye result. (**2**) Light microscopy image of an individual fiber of said strand, verifying the consistent dye result throughout the fiber. (**3**) SEM image of hair surface of said strand, showing cuticle layers with uneven precipitation of crystals. (**4**) EDX mapping of Ag and Cl on hair surface identifying the spatial proximity of the two elements on the hair surface and the unevenness of their distribution compared to the consistent red dye. (**B**) (**1**) Photograph of dye outcome of strand treated with 1% RE at pH 3 adjusted with CA and subsequent treatment with 1% AgNO_3_, depicting a consistently red dye result. (**2**) Light microscopy image of an individual hair fiber of said strand, verifying the consistent dye result throughout the fiber. (**3**) SEM image of hair surface of said strand, showing several layers of cuticle tightly fit but with no precipitate.

Yak hair strands treated according to the two-step method, first with 1% RE suspension and subsequently with 1% AgNO_3_, displayed a consistent red dye result after completion of dye development, regardless of the acid used to adjust the pH value Figure 1A(1,2),B(1,2). Additionally, the microscopic images revealed cuticle layers tightly attached to the fiber, suggesting that the dyeing conditions did not visibly disrupt the fiber surface.

Investigation of particles assigned to dyeing species on the hair surface using SEM revealed an irregular deposition of precipitate on the strand that was treated with 10% HCl (Figure 1A(3)). The dispersion of the grayish solid did not align with the evenness of the dye result. Results from EDX color mapping for chemical characterization of the hair surface showed a high spatial proximity of Ag and Cl promoting the assumption of AgCl deposition (Figure 1A(4)). In comparison, the hair strand that was adjusted to pH 3 using CA did not show a grayish precipitate on the surface in SEM (Figure 1B(3)), yet the strand exhibited a bright red dye result. In addition, SEM images affirmed the tight fit of the cuticle layers, although smaller fragments of cuticular cells were depicted (Figure 1A(3)).

**Figure 2:**
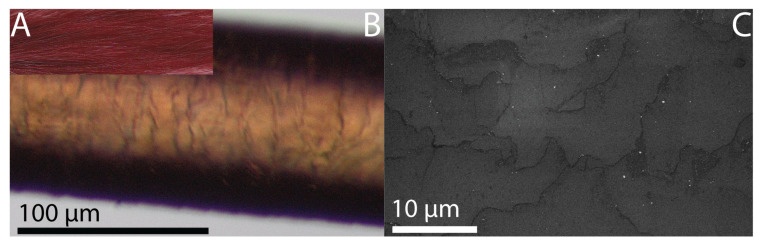
(**A**) Photograph of dye outcome of strand treated with 1% RE at pH 3 adjusted with 10% HCl and subsequent treatment with 1% AgNO_3_ and washed 24 times, still depicting an even red dye result. (**B**) Light microscopy image of an individual hair fiber of said strand, magnified 10-fold, showing cuticle layers and verifying the consistently red dye result throughout the hair fiber. (**C**) SEM image of hair surface of said strand showing several layers tightly fit to the cuticle, but no precipitate.

Washing the HCl-treated strand 24 times did not remove the uniform red dye result from the strand (Figure 2A,B). In contrast, the grayish precipitate on the surface of the hair fiber was taken away, verified by SEM (Figure 2C). This supports the assumption that AgCl deposited on the hair surface was not responsible for the observed dyeing outcome, indicating that the primary source of coloration originated from within the inner regions of the hair fiber. Additionally, the wash fastness testing had no visible impact on the quality of the cuticle since the layers remained tightly fit to the fiber.

### 2.2. Investigations of Hair Microtome Cuts

**Figure 3:**
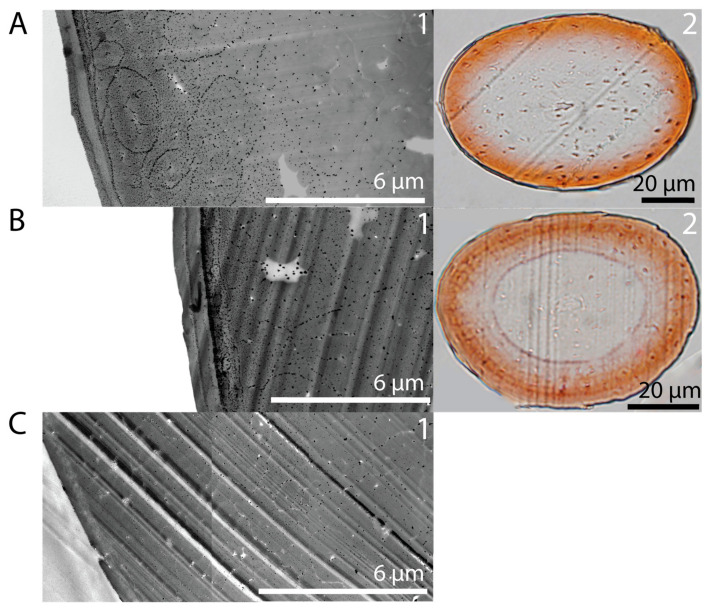
(**A**) TEM image of strand treated with 1% RE at pH 3 adjusted with 10% HCl and subsequent treatment with 1% AgNO_3_ (**1**) and microtome cut of said strand under light microscope (**2**). Particle concentration decreases from the surface into the fiber. The penetration depth of particles aligns with the microscopic image. (**B**) TEM image of strand treated with 1% RE at pH 3 adjusted with HCl (10%) and subsequently treated with 1% AgNO_3_ and washed 24 times (**1**), and microtome cut of said strand under light microscope (**2**). The strand shows a more consistent distribution of particles that is assumed to persist, reaching deeper into the fiber, and shows indentations resulting from the cutting process. (**C**) TEM image of strand treated with 1% RE at pH 3 adjusted with CA and subsequent treatment with 1% AgNO_3_ (**1**). Small particles are depicted uniformly distributed pointing inwards to the hair fiber.

Investigation of dyeing species inside the hair cortex using TEM of microtome cuts from strands treated first with 1% RE suspension, then acidified with 10% HCl and 1% AgNO_3_, showed small, roughly spherical particles inside the hair cuticula and cortex (Figure 3A(1)).

The concentration of the particles in unwashed strands was highest within the first 7.5 µm into the hair fiber. With the increase in penetration depth, a decreasing concentration of particles was observed. No particles were detected close to the medulla—the center of the hair fiber. Comparison with a light microscopy image of a treated strand showed a similar distribution of the red dye inside the cortex (Figure 3A(2)). The average penetration depth of the red dye circle was in line with the penetration depth of the particles.

TEM results for a treated strand that was washed 24 times showed a more in-depth diffusion of particles (Figure 3B(1)). Furthermore, the particles were more evenly distributed inside the cuticula and cortex pointing inwards into the hair fiber within the first 10 µm. The light microscopy image of a treated and washed strand (Figure 3B(2)) verified the red dye at the lower-lying layers of the hair cortex. Again, the average penetration depth of the red dye circle was in line with the penetration depth of the particles found in TEM. By contrast, TEM results for a strand treated with CA during the dyeing process showed a more uniform distribution of particles that overall reached deeper into the cortex (>10 µm) even without washing. Thus, the particles responsible for the dye outcome were rooted inside the hair.

**Figure 4:**
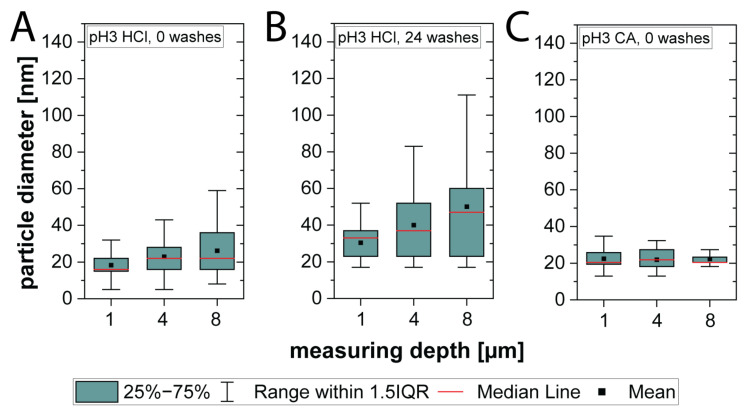
Box and whisker plots of particle diameter of nanoparticles at 1, 4 and 8 µm penetration depth into a hair fiber. Enlarged versions of Figure 3 were taken for measurement. (**A**) Number of particles (n = 67) from a strand initially treated with 1% RE at pH 3 HCl followed by 1% AgNO_3_, after 0 washes. Average size and size distribution of particles increase pointing inwards into the hair fiber. (**B**) Number of particles (n = 67) from a strand initially treated with 1% RE at pH 3 HCl followed by 1% AgNO_3_, after 24 washes. Average size and size distribution of particles increase pointing inwards into the hair fiber. (**C**) Number of particles (n = 30) from a strand initially treated with 1% RE at pH 3 CA followed by 1% AgNO_3_. Average size and size distribution of particles are consistent throughout the hair fiber. (IQR = interquartile range.) All mean and median values as well as a histogram for size distribution can be found in Tables S1 and S2 as well as Figure S1 in SI.

Particle size and size distribution inside the hair fiber were characterized using enlarged versions of TEM images from Figure 3. The hair fiber treated with 1% RE suspension acidified with 10% HCl revealed a wide range of particle sizes for both unwashed and washed hair strands that increased with the penetration depth, whereas the particle size for strands acidified with CA remained constant (Figure 4).

In an unwashed hair strand treated with a 1% RE suspension at pH 3 and acidified with 10% HCl, close to the surface (1 µm penetration depth), the middle 50% (blue box) of particle diameters ranged between approx. 15 and 20 nm (Figure 4A), with an average particle diameter of 18.5 ± 6.6 nm. The median value (red line) indicates that half of the particles were even smaller than 16.2 nm. At 4 µm penetration depth, the average particle size increased to 23.0 ± 8.9 nm and further to 26.3 ± 13.7 nm at 8 µm depth. At penetration depths of 4 and 8 µm, the particle size distribution broadened, indicating a wider spectrum of particle diameters. This dispersion was further substantiated by a corresponding increase in the interquartile range (IQR), reflecting enhanced variability. The middle 50% of particle diameters ranged between 16 and 28 nm at 4 µm penetration depth and between 16 and 36 nm at 8 µm. Yet, the median value for those size diameters did not increase (21.6 nm at 4 µm and 22.3 nm at 8 µm), indicating that at 8 µm, more particles with bigger diameters were observed.

The broadening of the particle size distribution became more pronounced after 24 washes (Figure 4B). In general, bigger particle diameters were observed compared to unwashed strands. Close to the surface of the hair fiber, at 1 µm penetration depth, the size for the middle 50% of particles lay between 23 and 37 nm, and an average particle diameter of 30.5 ± 12.4 nm was observed. Deeper inside the hair fiber, at a penetration depth of 4 µm, the size ranged from 23 to 52 nm (average diameter: 40.0 ± 17.4 nm) that stretched further up to 23–60 nm at 8 µm (average diameter: 50.2 ± 24.1 nm). The increased IQR reflects this variability and demonstrates that particle diameters surpassed 100 nm, even extending beyond the nanoscale at 8 µm.

Smaller particles sized < 5 nm were confirmed inside the hair as well, based on TEM images of ground hair (Figure S2 and Table S3). Thus, more particles might be located inside the hair cortex, contributing to the dye result. Due to limitations in resolution (5 nm at 0 washes and 16 nm at 24 washes), these particles could not be depicted in the TEM images of microtome cuts. However, close-up images of microtome cuts (0 washes) are included in SI (Figure S3), illustrating nanoparticles ≤ 10 nm.

In contrast, in a strand that underwent a treatment with 1% RE acidified to pH 3 with CA, particles maintained the same average size in all three layers of penetration investigated (1 µm: 22.5 ± 6.7 nm, 4 µm: 21.9 ± 5.8 nm, 8 µm: 22.3 ± 4.9 nm) (Figure 4C). Median values behaved accordingly. In fact, the size distribution, including IQR, decreased with penetration depth of the particles. Histograms, further visualizing the size distribution of the particles in each formulation, can be found in Figure S1.

Interestingly, the overall visual dye results of the strands were similar, regardless of the acid applied and the depicted changes in particle size. Furthermore, no noticeable change in color was observed despite washing and blow-drying (50 °C).

**Figure 5:**
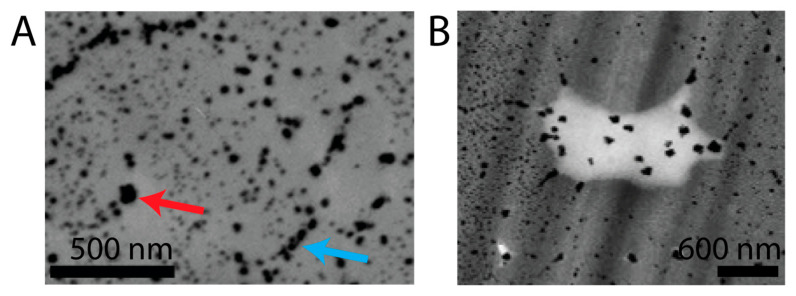
(**A**) Excerpt of TEM image of strand treated with 1% RE at pH 3, adjusted with HCl (10%), and subsequent treatment with 1% AgNO_3_. Blue arrow marks approximated NPs; red arrow marks agglomerated or merged NPs. (**B**) Excerpt of TEM image of strand treated with 1% RE at pH 3 adjusted with 10% HCl and subsequent treatment with 1% AgNO_3_, and which was washed 24 times. Incidental cavity in hair matrix holds larger particles than denser surrounding hair matrix.

Close-ups from TEM images of microtome cuts from strands acidified to pH 3 with 10% HCl verified different stages of particle agglomeration (Figure 5A), including particles being in proximity (blue arrow) as well as bigger particles, potentially after completion of the coalescing process (red arrow). Additionally, size distribution of 19 particles inside an incidental cavity in the hair cortex of a washed strand (Figure 5B) revealed an average particle diameter of 94.6 ± 27.6 nm, suggesting that the growth of particles is controlled by the density of the surrounding hair matrix. Those NPs mostly lost their spherical shape, becoming more randomly shaped as a consequence of the coalescing process.

**Figure 6:**
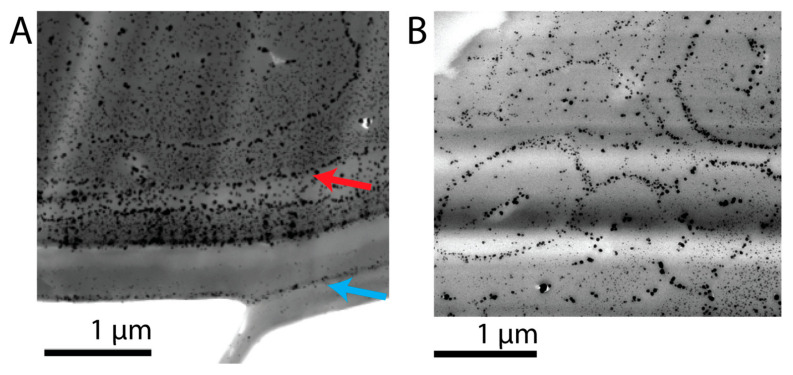
TEM images of strand treated with 1% RE at pH 3 adjusted with 10% HCl and subsequent treatment with 1% AgNO_3_ after zero washes. (**A**) Particles show a lamellar distribution along the cuticular layers (blue and red arrow) and accumulate within the hair cortex. (**B**) Particles accumulate inside the hair cortex in the cortex–cortex CMC that surrounds the cortical cells.

Varying distribution patterns of the particles were observed, depending on the location within the hair fiber (Figure 6). Within the cuticle, a few particles aggregated alongside a thin line at the upper section of each layer, highlighting the stratified shape of the cuticle (Figure 6A, blue arrow). Additionally, a high concentration of particles was depicted alongside the cuticula–cortex CMC, which is the crossover to the cortex (red arrow). An overall orientation of the particles towards the cortex of the hair was observed, resulting in a circular distribution of the particles (Figure 6B). Particles aggregated alongside the cortex–cortex CMC that surrounds the cortical cells.

## 3. Discussion

The coloring agent was observed using light and electron microscopy on microtome cuts. The appearance of the color was congruent with the appearance of particles and was thus attributed to the formation of AgNPs inside the hair fiber. The quasi-spherical shape observed in the AgNPs was characteristic of plant-mediated synthesis, reflecting the typical outcome when plant compounds are used as reducing and capping agents in synthesis [7,8,19,50]. Metal nanoparticles naturally adopt a cubic crystal structure [51] and tend to grow isotropically in the absence of surface-active ligands such as oleylamine or trioctylphosphine (TOP). These ligands, which reduce the surface energy of specific facets, are required for anisotropic growth (e.g., rod-shaped particles). As no such ligands were used in this study, the observed spherical morphology is typical for unmodified AgNPs. The observed increase in particle diameter from the surface inwards in strands that were treated with 1% RE suspension acidified to pH 3 with 10% HCl is ascribed to the growth and aggregation behavior of silver nanoparticles. According to Ostwald ripening, smaller nanoparticles dissolve in favor of the growth of larger NPs [52,53]. Since this theory was proposed, much research has been performed in this field; hence a more recent approach suggests the aggregation and coalescence of small NPs and their subsequent growth [54,55].

It is further known that reducing and capping agents have a major impact on the size and shape of the resulting NPs. Small concentrations of chloride ions enhance the agglomeration process of AgNP according to Li et al. [24,25]. Herein, the electrolyte interacts with silver ions released from the NPs’ surfaces, thus decreasing the electrostatic energy barrier that hinders the NPs from agglomeration. El Badawy et al. [56] found that the addition of chloride results in the formation of negatively charged AgCl colloids that subsequently interact with silver cations from the adjacent NPs, promoting coalescence.

Our findings show that the number of particles for strands acidified to pH 3 with 10% HCl decreased into the fiber and only a few small particles were detected at an 8 µm penetration depth, whereas the diameter of particles increased. This observation could thus be attributed to the presence of chloride in the system, promoting the agglomeration of NPs. Furthermore, close-ups from TEM images capture different stages of particle growth, supporting the hypothesis of agglomeration and the coalescing process of smaller particles. Additionally, the occurrence of relatively bigger particles in washed strands might derive from repeated contact with water, enhancing the mobility of particles towards one another, thus promoting the aforementioned processes. However, a chloride-dependent influence on the reaction time in the formation of particles, as reported by King et al. [57], was not observed. The color development progressed regardless of the additive [56,57]. The penetration pattern of particles for pH 3 HCl changed after 24 washes, indicating an increase in penetration depth in accordance with the penetration depth of the red dye impression, linking the dye result to the presence of particles.

Citrate can act as both a reducing and capping agent for silver ions, whereas its reductive properties are described as rather weak and mainly relevant at higher temperatures [58,59]. Its capability to act as a capping agent is attributed to carboxyl groups with free electron pairs resulting in electrostatic stabilization that can induce a repelling of particles and thus more uniformly shaped NPs [23,56,58,60,61]. Particles in fibers that were acidified to pH 3 with CA during the dyeing process maintained their average size in all three penetration depths investigated and their size distribution even decreased. This observation could thus be attributed to the presence of citrate in the system, which promotes the repelling of the particles. In addition, a narrow size distribution implies more brilliant colors, which is an advantage for hair dyeing purposes.

In this study, we observed a red color, regardless of the application of either CA or HCl and the size distribution of particles [62,63]. No change in color was detected after 24 washes, including blow-drying with a commercial hair dryer, demonstrating long-term color fastness, a critical performance metric for hair dye systems. Compared to bulk silver, AgNPs exhibit a reduced melting point [64,65], making them more susceptible for heat-induced sintering, thus leading to the formation of larger particles that could potentially alter the dye impression [66,67,68]. In our experiments as well as our previous study [21], the temperature of the hair surface did not exceed 50 °C and no observable color change or NP instability was detected, indicating that the drying process did not lead to significant thermal degradation. These findings are in accordance with the literature, where even nanoparticles with a diameter of approximately 2.5 nm still exhibit melting temperatures in the range of 587–607 °C (860–880 K) [65]. This melting point significantly exceeds the maximum temperatures expected during blow-drying procedures. The thermal stability of AgNPs may help maintain color integrity over time and may ensure that they retain their beneficial effects throughout the typical lifespan of the dye. However, further research is necessary to evaluate the effect of thermal styling tools such as straightening irons on long-term color stability, given that these tools simultaneously apply direct heat higher than 200 °C and pressure to the hair fiber.

The challenges in the present work lie in the hair matrix structure, complicating several commonly used analytical methods to investigate NPs in vitro, and the fact that, to date, not much research has been performed investigating nanoparticles in a solid organic medium [32,33]. Since the amount of silver is relatively small compared to the amount of hair matrix (max. 3 weight%) [21], potential signals for NPs resulting from solid-phase NMR (HR-MAS) stepped back behind the background noise of the hair matrix. Due to unsuccessful outcomes of the experiments, FT-IR-spectroscopy analyses of ground or whole hair fibers could not provide further information regarding the interaction of polyphenolic compounds with keratin moieties to verify potential oxidation products. The large ratio between the hair matrix and polyphenols did not allow us to distinguish between the two components and thus prevented the identification of polyphenols in the sample. Additionally, the cylindrical shape of the hair and its irregular surface complicated optical analytics by reflecting and dispersing the light rather than absorbing. Investigations using confocal laser spectroscopy revealed changing densities of the hair matrix within a few micrometers of distance, disrupting the recording of a correct baseline. Additionally, charge effects of the hair during SEM measurements complicated imaging.

The most suitable analytical method was TEM imaging of microtome cuts. The “snapshot” allowed the observation of the characteristic distribution and accumulation patterns of the particles with spatial resolution. Nevertheless, the preparation of the images was challenging since the particles were embedded within the organic matrix. This embedding caused a blurring of the nanoparticle boundaries, which in some cases gave the appearance of a decreased contrast during size measurements.

Whereas an overall orientation of particles towards the hair cortex was observed, the cuticle showed accumulation of particles along the exocuticle as well as cuticle–cuticle CMC [47]. Due to the limited resolution of the images, a further differentiation between the layers could not be performed. Within the cortex, particles were distributed alongside the cortex–cortex CMC surrounding the cortex cells [40,69,70]. For sheep-wool fibers it was found that the cortex–cortex CMC contained more sulfur moieties than the cuticle–cuticle CMC, especially in terms of di-sulfide moieties, depicting a potentially favorable environment for silver [47]. Charge differences between broken di-sulfidic bonds and silver cations on the surface of AgNPs might have further promoted the attraction towards the cortex [71,72]. Additionally, the less-tightly fit CMC might have offered more space for particles to grow than the dense macrofibrils. Larger particles found in cavities of the hair fiber support this assumption. However, particles smaller than 5 nm could be proven in ground hair as well; thus smaller particles are supposedly able to enter the macrofibrils [45,47].

Despite their promising application potential as a hair dye, AgNPs present a toxicological risk depending on their route of exposure. Principally, intact skin is found to be an efficient barrier against systemic AgNP uptake [73], even though some studies report skin sensitization or inflammation when AgNPs are applied to the skin [74,75], presumably resulting from a subsequent silver ion release [76]. It is further shown that capped spherical AgNPs of 25 nm diameter could penetrate the epidermis in humans and that hair shafts play a crucial role for the penetration process, given that they constitute a hole for accumulation of particles [77,78]. Despite differing opinions on dermal toxicity, in the context of this work, both the mentioned particle size and their proximity to hair shafts are addressed, highlighting the need for further investigation into long-term outcomes.

## 4. Materials and Methods

### 4.1. Materials

Yak hair tresses were obtained from Kerling International Haarfabrik GmbH, Backnang, Germany, with a total length of 11.8 cm of which 7.5 cm included hair fibers and the weight of fibers was 0.7 g. Ultra-pure water (UPW) was used throughout all experiments. The purification was conducted using the Arium Pro Ultra-Pure Water System from Sartorius AG, Göttingen, Germany. All investigations were conducted using a hydro-alcoholic extract powder of aerial flowering parts of *Reseda luteola* L., kindly provided by Couleur de Plantes (Rochefort, France). The plants were harvested in France in August 2013 and extracted in November 2022. Silver nitrate (AgNO_3_, CAS: 7761-88-8) was purchased from Carl Roth, Karlsruhe, Germany. Citric acid anhydrous (C_6_H_8_O_7_, CAS: 77-92-9) was purchased from Inter-Harz GmbH, Klein-Offenseth-Sparrieshop, Germany, and hydrochloric acid (HCl 37%, CAS: 7647-01-0) was purchased from VWR International GmbH, Darmstadt, Germany. Aqueous dilutions to concentrations of HCl of 10% or sulfuric acid of 20% were freshly prepared in the laboratory. The additives used were of analytical grade.

### 4.2. Pre-Treatment of Hair Strands

Before dyeing, the hair strands were pre-cleansed according to the following method: The strands were wetted at room temperature (r.t.) with a total UPW flow of 25 mL s^−1^ and placed in 20 mL/strand of an aqueous 12.5% sodium laureth sulfate (SLES, Henkel AG & Co. KGaA, Düsseldorf, Germany) solution adjusted to pH 4.5 using 20% sulfuric acid for 30 min. It was ensured that the strands were fully covered in the cleansing bath. The strands were subsequently rinsed twice with UPW at a total flow of 25 mL s^−1^, combed 20 times and air-dried. The strands were used without any further pre-treatment.

### 4.3. General Dyeing Method Using Reseda luteola L. and AgNO_3_

The dyeing was conducted in a two-step process, as described in our previous publication [21]. At first, 500 mg RE was suspended in 50 mL of UPW to obtain an aqueous RE suspension (1% *w*/*v*) with a natural pH of 5.3. Subsequently, the pH was adjusted to pH 3 using either 10% HCl (aq.) or citric acid. The strands were stirred in the suspension at r.t. for 30 min. Afterwards, the strands were rinsed at r.t. with a total UPW flow of 25 mL s^−1^, combed 20 times and blow-dried with a commercially available blow-dryer for 60 s at a distance of 20–25 cm from the hair dryer. The temperature of the hair surface did not exceed 50 °C, determined using the Traceable^®^ Infrared Thermometer (VWR International GmbH, Darmstadt, Germany).

In a second step, 500 mg silver nitrate per strand was dissolved in 50 mL UPW (1% *w*/*v*) and the pre-treated strands were stirred at r.t. for 30 min under exclusion of light. The strands were again rinsed, combed and blow-dried as described above. Subsequently, the strands were irradiated under a PAR38 full-spectrum plant-lamp (Ross Gesundes Licht, Hamburg, Germany), emitting UV/Vis radiation within 390–780 nm. The distance between the light source and strands was 35 cm and the strands were kept inside the closed chamber for at least 5 h.

### 4.4. Method of Wash Fastness Testing

The strands were wetted at r.t. with a total UPW flow of 25 mL s^−1^ and washed up to 24 times (4 wash cycles) using the commercially available Schauma 7 Kräuter Shampoo (Schwarzkopf, Düsseldorf, Germany). One wash cycle comprised 6 washes with 0.5 g shampoo. The shampoo was massaged into the strands ten times from bond to tip (duration: 40 s). Afterwards, the strands were rinsed with a total water flow of 25 mL s^−1^ and combed (duration: 45 s). The procedure was repeated five times. After the last rinse, the strands were blow-dried for 60 s at a distance of 20–25 cm from the hair dryer. The temperature of the hair surface did not exceed 50 °C, determined using the Traceable^®^ Infrared Thermometer (VWR International GmbH, Darmstadt, Germany).

### 4.5. Analytics of Hair Fiber Surface

#### 4.5.1. Light Microscopy Images of Hair Surface

Images of the hair surface were taken with an Olympus BS53 equipped with an Olympus U-TV1X-2 camera (both Olympus Deutschland GmbH, Hamburg, Germany) and objectives with several magnifications. OLYMPUS Stream Start 2.5 software (Olympus Soft Imaging Solutions GmbH, Münster, Germany) was used for operating the camera.

#### 4.5.2. SEM Images and EDX Mapping of Hair Surface

SEM images of hair fibers were conducted on the field emission scanning electron microscope Zeiss LEO Gemini 1550 equipped with an Inlens detector. EDX was conducted using a Silicon-Drift-Detector (SDD) Ultim Max 100 (Carl Zeiss Microscopy Deutschland GmbH, Oberkochen, Germany).

### 4.6. Analytics of Hair Microtome Cuts

#### 4.6.1. Light Microscopy

Cross-sections of hair fibers were cut at the Henkel AG & Co KGaA site (Düsseldorf, Germany) using Epredia NX70 with Vakutom (Leica Biosystems Nussloch GmbH, Nussloch Germany). The hair samples were embedded in Tissue Freezing Medium (Leica) and frozen to −50 °C. Subsequently, the fibers were cut at −16 °C and a knife temperature of −23 °C to a thickness of 3 µm. The cuts were finally fixed between the glass plates.

Microscopic images were taken using an Olympus BX51 fluorescence microscope and Olympus DP71 digital microscope camera (Olympus Europa SE & Co. KG, Hamburg, Germany).

#### 4.6.2. Transmission Electron Microscopy of Microtome Cuts

Hair fibers were embedded in an Agar resin (Plano GmbH, Wetzlar, Germany) and cut using the Ultramicrotome Ultracut E (Reichert-Jung) equipped with a diamond knife Ultra 45 (Diatome, Quakertown, PA, USA) to a thickness of ca. 330 nm. The cuts were placed on 400 mesh Cu TEM grids, G2400C (Plano GmbH, Wetzlar, Germany), and analyzed with the Tecnai 10 (Philips) at an accelerating voltage of 60 kV equipped with a TEM camera Tengra (Olympus Soft Imaging Solutions GmbH, Münster, Germany).

#### 4.6.3. Determination of Particle Size on TEM Images of Microtome Cuts

The determination of the particle diameter was conducted using Adobe Photoshop, Version: 23.0.1 (Adobe Inc., San José, CA, USA). A 1 × 1 µm sized area was selected, of which the center was at a penetration depth of either 1, 4 or 8 µm into the hair fiber starting from the hair surface. Inside the selected area, 67 particles for strands acidified with 10% HCl and 30 particles for strands acidified using CA, respectively, were determined visually using the Photoshop Ruler tool and correlating pixel length to the physical length (nm). The data were collected for statistical analysis.

### 4.7. Transmission Electron Microscopy of Ground Hair Strands

Hair fibers were ground into a powder using a Pulverisette 23 ball mill (Fritsch GmbH, Idar-Oberstein, Germany) equipped with a zirconium oxide grinding bowl and balls, respectively. The runtime was 2 × 5 min with a cool-down time of 2–3 min in between and a frequency of 50 per second.

To investigate the size of NPs on ground hair samples, transmission electron microscopy (TEM) was performed using the JEOL JEM 1011 at an acceleration voltage of 100 kV (JEOL Germany GmbH, Freising, Germany). Samples were prepared by drop coating the hair suspensions on carbon-coated copper TEM grids.

### 4.8. Statistical Analysis

Statistical analysis was performed using IBM SPSS Statistics Version 29.0 (IBM Corp., Armonk, NY, USA) and OriginPro 2022 (OriginLab Corp. Northampton, MA, USA). Descriptive statistics, comprising mean value, standard deviation, median and values for the interquartile range were collected.

## 5. Conclusions

To the best of our knowledge, this study is the first to demonstrate the presence of silver nanoparticles (AgNPs) inside hair fibers for dyeing applications, highlighting the potential of metal-nanoparticle-based hair dyes. Building upon our previous study [21], we were able to correlate the penetration depth of the red dye with the distribution of nanoparticles observed in TEM images. This allowed us to attribute the dyeing effect to the presence of particles inside the hair fiber, thereby substantiating the observed high wash fastness.

The imaging methods TEM and SEM delivered important information regarding the location of the dyeing species straight to the point inside the hair fiber and highlighted the size and aggregation behavior depending on the capping agent and consequential interactions with the surrounding organic medium. The aggregation in presumably sulfur-rich domains observed inside the hair fiber was related to the affinity of silver ions on the NP’s surface towards sulfur. Dye uniformity was evaluated using light microscopy and was comparable regardless of the addition of chloride or citrate, even though the particle size and distribution behavior differed. More attention is needed regarding the surface composition of the NPs inside the hair fiber to achieve an even deeper understanding of the interaction of silver, polyphenols and additives, also in respect of interactions with keratinous material. At this point, the imaging methods applied are useful tools for the analysis of particle-based hair dyes. Yet, difficulties, such as background noise derived from the hair material and resolution of images, remain, affecting the applicability of methods like Raman spectroscopy, HR-MAS or confocal spectroscopy, as discussed.

For now, a red shade with a high wash fastness was realized. The control over the particle size remained limited; however, the synthesis of nanoparticles with varied sizes and shapes offered a promising route to expand the shade portfolio, though size control as well as control over their retention within hair fibers remains to be thoroughly evaluated. To date, no environmental impacts, such as UV-exposure, have been assessed, but this needs consideration for the development of a final dye product.

## Data Availability

The original contributions presented in this study are included in the article/Supplementary Materials. Further inquiries can be directed to the corresponding author(s).

## Supplementary Materials

The following supporting information can be downloaded at: https://www.mdpi.com/article/10.3390/molecules30163446/s1, Table S1: Average nanoparticle size (mean ± 2 SD) for strands adjusted to pH 3 using HCl in unwashed and washed hair, as well as strands adjusted to pH 3 using CA, each at 1, 4 and 8 µm penetration depth. Table S2: Median values for strands adjusted to pH 3 using HCl in unwashed and washed hair, as well as strands adjusted to pH 3 using CA, each at 1, 4 and 8 µm penetration depth. Figure S1: (A) Histograms depicting particle distribution, mean particle size (mean ± SD) and sample size (n). Figure S2: (A) Excerpt of TEM image of ground hair strand treated with 1% RE at pH 3 adjusted with HCl (10%) and subsequent treatment with 1% AgNO_3_. Numbers highlight the depicted particles. Table S3: Particle diameters from Figure S1. Figure S3: Excerpts of TEM images of microtome cut treated with 1% RE at pH 3 adjusted with 10% HCl and subsequent treatment with 1% AgNO_3_ illustrating nanoparticles ≤ 10 nm.

## Abbreviations

The following abbreviations are used in this manuscript:

UPW	Ultra-Pure Water
RE	*Reseda luteola* L. Extract
HCl	Hydrochloric acid
CA	Citric Acid
XRD	X-Ray Powder Diffractometry
TEM	Transmission Electron Microscopy
SEM	Scanning Electron Microscopy
AgNP	Silver Nanoparticle
EDX	Energy-Dispersive X-Ray Spectroscopy
r.t.	Room Temperature
TOP	Trioctylphosphine
Å	Ångström
SLES	Sodium Laureth Sulfate
SD	Standard Deviation
IQR	Interquartile Range
CMC	Cell Membrane Complex
FT-IR	Fourier-Transformed Infrared Spectroscopy
HR-MAS	High-Resolution Magic Angle Spinning
NMR	Nuclear Magnetic Resonance Spectroscopy
h	Hours

## References

[1] Biertümpfel A., Wurl G., Bechtold T., Mussak R. (2009). Dye Plants in Europe. Handbook of Natural Colorants.

[2] Sankar J., Sawarkar S., Malakar J., Singh Rawa B., Asif Ali M. (2017). Mechanism of Hair Dying and Their Safety Aspects: A Review. Asian J. Appl. Sci..

[3] Tkaczyk A., Mitrowska K., Posyniak A. (2020). Synthetic Organic Dyes as Contaminants of the Aquatic Environment and Their Implications for Ecosystems: A Review. Sci. Total Environ..

[4] Cui H., Cai R., Hua Z., Tang Y. (2023). Plant Colorants for Natural Hair Coloration: Dyeing Optimization and Photostability Assessment. Sustain. Chem. Pharm..

[5] Ali S., Maqbool M., Hussain M.T. (2022). Efficacy of Some Plants Extracts for Natural Dyeing of Human Hair. J. Nat. Fibers.

[6] Akhtar M.S., Panwar J., Yun Y.-S. (2013). Biogenic Synthesis of Metallic Nanoparticles by Plant Extracts. ACS Sustain. Chem. Eng..

[7] Rizwana H., Alwhibi M.S., Al-Judaie R.A., Aldehaish H.A., Alsaggabi N.S. (2022). Sunlight-Mediated Green Synthesis of Silver Nanoparticles Using the Berries of Ribes Rubrum (Red Currants): Characterisation and Evaluation of Their Antifungal and Antibacterial Activities. Molecules.

[8] Filip G.A., Moldovan B., Baldea I., Olteanu D., Suharoschi R., Decea N., Cismaru C.M., Gal E., Cenariu M., Clichici S. (2019). UV-Light Mediated Green Synthesis of Silver and Gold Nanoparticles Using Cornelian Cherry Fruit Extract and Their Comparative Effects in Experimental Inflammation. J. Photochem. Photobiol. B.

[9] Prathna T.C., Raichur A.M., Chandrasekaran N., Mukherjee A. (2014). Sunlight Irradiation Induced Green Synthesis of Stable Silver Nanoparticles Using Citrus Limon Extract. Proc. Natl. Acad. Sci. India Sect. B Biol. Sci..

[10] Mathew S., Prakash A., Radhakrishnan E.K. (2018). Inorganic and Nano-Metal Chemistry Sunlight Mediated Rapid Synthesis of Small Size Range Silver Nanoparticles Using Zingiber Officinale Rhizome Extract and Its Antibacterial Activity Analysis. Inorg. Nano-Metal. Chem..

[11] Singh J., Singh Dhaliwal A. (2018). Novel Green Synthesis and Characterization of the Antioxidant Activity of Silver Nanoparticles Prepared from Nepeta Leucophylla Root Extract. Anal. Lett..

[12] Sooraj M.P., Nair A.S., Vineetha D. (2021). Sunlight-Mediated Green Synthesis of Silver Nanoparticles Using Sida Retusa Leaf Extract and Assessment of Its Antimicrobial and Catalytic Activities. Chem. Pap..

[13] Silva Brito R., João Bebianno M., Rocha T.L. (2024). Plant-Based Silver Nanoparticles Ecotoxicity: Perspectives about Green Technologies in the One Health Context. Crit. Rev. Environ. Sci. Technol..

[14] Mohanta Y.K., Panda S.K., Bastia A.K., Mohanta T.K. (2017). Biosynthesis of Silver Nanoparticles from Protium Serratum and Investigation of Their Potential Impacts on Food Safety and Control. Front. Microbiol..

[15] Melo M.J., Bechthold T., Mussak R. (2009). History of Natural Dyes in the Ancient Mediterranean World. Handbook of Natural Colorants.

[16] Ferreira E.S.B., Hulme A.N., Mcnab H., Quye A. (2004). The Natural Constituents of Historical Textile Dyes. Chem. Soc. Rev..

[17] Mesrar F.E., Tachallait H., Bougrin K., Benhida R. (2024). Ultrasound-Assisted Extraction of Vegetable Dyes and Mordants from Wool Dyed with Curcuma Longa and Reseda Luteola. Ind. Crops Prod..

[18] Safapour S., Mazhar M., Abedinpour S. (2023). Color Shade Extension of Reseda Luteola L. Natural Colorant on Wool Textiles via Binary Combination of Metal Salts: Colorimetric and Fastness Studies. Fibers Polym..

[19] Boroumand M.N., Montazer M., Dutschk V. (2013). Biosynthesis of Silver Nanoparticles Using Reseda Luteola L and Their Antimicrobial Activity. Ind. Textila.

[20] Jafari R., Gharanjig K., Hosseinnezhad M. (2023). Substitution of Metal Ion Mordant with Biomordants: Effect on Color and Fastness of Reseda Dyed on Wool Yarns. J. Text. Inst..

[21] Hachmann J.K., Sauler J.M., Ruhmlieb C., Vill V., Straske F. (2025). Red Era: Dyeing Yak Hair Fibres Red with in Situ Generated Silver Nanoparticles Using Silver Nitrate and a Flavonoid-containing Plant Extract. Color. Technol..

[22] Sidhu A.K., Verma N., Kaushal P. (2022). Role of Biogenic Capping Agents in the Synthesis of Metallic Nanoparticles and Evaluation of Their Therapeutic Potential. Front. Nanotechnol..

[23] Henglein A., Giersig M. (1999). Formation of Colloidal Silver Nanoparticles: Capping Action of Citrate. J. Phys. Chem. B.

[24] Li X., Lenhart J.J., Walker H.W. (2012). Aggregation Kinetics and Dissolution of Coated Silver Nanoparticles. Langmuir.

[25] Li X., Lenhart J.J., Walker H.W. (2010). Dissolution-Accompanied Aggregation Kinetics of Silver Nanoparticles. Langmuir.

[26] Siakavella I.K., Lamari F., Papoulis D., Orkoula M., Gkolfi P., Lykouras M., Avgoustakis K., Hatziantoniou S. (2020). Effect of Plant Extracts on the Characteristics of Silver Nanoparticles for Topical Application. Pharmaceutics.

[27] Mittal A.K., Chisti Y., Banerjee U.C. (2013). Synthesis of Metallic Nanoparticles Using Plant Extracts. Biotechnol. Adv..

[28] Montes-Hernandez G., Di Girolamo M., Sarret G., Bureau S., Fernandez-Martinez A., Lelong C., Eymard Vernain E. (2021). In Situ Formation of Silver Nanoparticles (Ag-NPs) onto Textile Fibers. ACS Omega.

[29] Rajput S.K., Singh M.K., Shakyawar D.B. (2025). Herbal Synthesis of Silver Nanoparticles for Improved Dyeing and UV Protection of Cotton Fabric. Text. Res. J..

[30] Shahid M., Cheng X.W., Tang R.C., Chen G. (2017). Silk Functionalization by Caffeic Acid Assisted In-Situ Generation of Silver Nanoparticles. Dye. Pigment..

[31] Sadeghi-Kiakhani M., Tehrani-Bagha A.R., Miri F.S., Hashemi E., Safi M. (2022). Eco-Friendly Procedure for Rendering the Antibacterial and Antioxidant of Cotton Fabrics via Phyto-Synthesized AgNPs With Malva Sylvestris (MS) Natural Colorant. Front. Bioeng. Biotechnol..

[32] Haveli S.D., Walter P., Patriarche G., Ayache J., Castaing J., Van Elslande E., Tsoucaris G., Wang P.-A., Kagan H.B. (2012). Hair Fiber as a Nanoreactor in Controlled Synthesis of Fluorescent Gold Nanoparticles. Nano Lett..

[33] Tang Y., Zhang Z., Yang S., Smith G.J., Liu L. (2021). Diatomite Encapsulated AgNPs as Novel Hair Dye Cosmetics: Preparation, Performance, and Toxicity. Colloids Surf. B Biointerfaces.

[34] Im D.S., Hong B.M., Kim M.H., Park W.H. (2020). Formation of Human Hair-Ag Nanoparticle Composites via Thermal and Photo-Reduction: A Comparison Study. Colloids Surf. A Physicochem. Eng. Asp..

[35] Deng D., Gopiraman M., Kim S.H., Chung I.M., Kim I.S. (2016). Human Hair: A Suitable Platform for Catalytic Nanoparticles. ACS Sustain. Chem. Eng..

[36] Gopiraman M., Deng D., Zhang K.-Q., Kai W., Chung I.-M., Karvembu R., Kim I.S. (2017). Utilization of Human Hair as a Synergistic Support for Ag, Au, Cu, Ni, and Ru Nanoparticles: Application in Catalysis. Ind. Eng. Chem. Res..

[37] De Cássia Comis Wagner R., Kunihiko Kiyohara P., Silveira M., Joekes I. (2007). Electron Microscopic Observations of Human Hair Medulla. J. Microsc..

[38] Solovan C., Doroftei F., Pinteala M., Chiriac A., Cristea C. (2015). Scanning Electron Microscopic Examination of the Hair Shaft Abnormalities in Netherton’s Syndrome. Int. J. Dermatol..

[39] Yang F.-C., Zhang Y., Rheinstädter M.C. (2014). The Structure of People’s Hair. PeerJ.

[40] Coroaba A., Chiriac A.E., Sacarescu L., Pinteala T., Minea B., Ibanescu S.-A., Pertea M., Moraru A., Esanu I., Maier S.S. (2020). New Insights into Human Hair: SAXS, SEM, TEM and EDX for Alopecia Areata Investigations. PeerJ.

[41] Kadir M., Wang X., Zhu B., Liu J., Harland D., Popescu C. (2017). The Structure of the ‘’amorphous” Matrix of Keratins. J. Struct. Biol..

[42] Essendoubi M., Andre N., Granger B., Clave C., Manfait M., Thuillier I., Piot O., Ginestar J. (2022). New Approach for Hair Keratin Characterization: Use of the Confocal Raman Spectroscopy to Assess the Effect of Thermal Stress on Human Hair Fibre. Int. J. Cosmet. Sci..

[43] Essendoubi M., Meunier M., Scandolera A., Gobinet C., Manfait M., Lambert C., Auriol D., Reynaud R., Piot O. (2019). Conformation Changes in Human Hair Keratin Observed Using Confocal Raman Spectroscopy after Active Ingredient Application. Int. J. Cosmet. Sci..

[44] Müllner A.R.M., Pahl R., Brandhuber D., Peterlik H., Lichtenegger H., Rennhofer H. (2020). Porosity at Different Structural Levels in Human and Yak Belly Hair and Its Effect on Hair Dyeing. Molecules.

[45] Gummer C.L. (2001). Elucidating Penetration Pathways into the Hair Fiber Using Novel Microscopic Techniques. J. Cosmet. Sci..

[46] Rust R.C., Schlatter H., Draelos Z.D. (2016). Hair Dyes. Cosmetic Dermatology: Products and Procedures.

[47] Robbins C.R. (2012). Chemical and Physical Behavior of Human Hair.

[48] Liu H.L., Zhao B.Y., Yu W.D. (2013). Structural Changes in Slenderized Yak Hair Induced by Heat-Humidity Conditions Using Raman Spectroscopy. J. Mol. Struct..

[49] Negri A.P., Cornell H.J., Rivett D.E. (1993). A Model for the Surface of Keratin Fibers. Text. Res. J..

[50] Raj S., Trivedi R., Soni V. (2022). Biogenic Synthesis of Silver Nanoparticles, Characterization and Their Applications—A Review. Surfaces.

[51] Sow C., Mettela G., Kulkarni G.U. (2020). Noble Metal Nanomaterials with Nontraditional Crystal Structures. Annu. Rev. Mater. Res..

[52] Dong Y., Zhang D., Li D., Jia H., Qin W. (2023). Control of Ostwald Ripening. Sci. China Mater..

[53] Ostwald W. (1897). Studien Über Die Bildung Und Umwandlung Fester Körper: 1. Abhandlung: Übersättigung Und Überkaltung. Z. Phys. Chem..

[54] Richards V.N., Rath N.P., Buhro W.E. (2010). Pathway from a Molecular Precursor to Silver Nanoparticles: The Prominent Role of Aggregative Growth. Chem. Mater..

[55] Zheng H., Smith R.K., Jun Y.W., Kisielowski C., Dahmen U., Paul Alivisatos A. (2009). Observation of Single Colloidal Platinum Nanocrystal Growth Trajectories. Science.

[56] El Badawy A.M., Luxton T.P., Silva R.G., Scheckel K.G., Suidan M.T., Tolaymat T.M. (2010). Impact of Environmental Conditions (PH, Ionic Strength, and Electrolyte Type) on the Surface Charge and Aggregation of Silver Nanoparticles Suspensions. Environ. Sci. Technol..

[57] King M.E., Kent I.A., Personick M.L. (2019). Halide-Assisted Metal Ion Reduction: Emergent Effects of Dilute Chloride, Bromide, and Iodide in Nanoparticle Synthesis. Nanoscale.

[58] Chadha R., Maiti N., Kapoor S. (2014). Reduction and Aggregation of Silver Ions in Aqueous Citrate Solutions. Mater. Sci. Eng. C.

[59] Jiang X.C., Chen C.Y., Chen W.M., Yu A.B. (2010). Role of Citric Acid in the Formation of Silver Nanoplates through a Synergistic Reduction Approach. Langmuir.

[60] Ranoszek-Soliwoda K., Tomaszewska E., Socha E., Krzyczmonik P., Ignaczak A., Orlowski P., Krzyzowska M., Celichowski G., Grobelny J. (2017). The Role of Tannic Acid and Sodium Citrate in the Synthesis of Silver Nanoparticles. J. Nanopart Res..

[61] Maccuspie R.I. (2011). Colloidal Stability of Silver Nanoparticles in Biologically Relevant Conditions. J. Nanopart Res..

[62] Al-Ghamdi H.S., Mahmoud W.E. (2013). One Pot Synthesis of Multi-Plasmonic Shapes of Silver Nanoparticles. Mater. Lett..

[63] Tang B., Li J., Hou X., Afrin T., Sun L., Wang X. (2013). Colorful and Antibacterial Silk Fiber from Anisotropic Silver Nanoparticles. Ind. Eng. Chem. Res..

[64] Luo W., Hu W., Xiao S. (2008). Size Effect on the Thermodynamic Properties of Silver Nanoparticles. J. Phys. Chem. C.

[65] Feng D., Feng Y., Yuan S., Zhang X., Wang G. (2017). Melting Behavior of Ag Nanoparticles and Their Clusters. Appl. Therm. Eng..

[66] Hu Y., Wang Y., Yao Y. (2023). Molecular Dynamics on the Sintering Mechanism and Mechanical Feature of the Silver Nanoparticles at Different Temperatures. Mater. Today Commun..

[67] Park H.J., Ryu K., Lee H.L., Moon Y.J., Hwang J.Y., Moon S.J. (2024). Physical Characteristics of Sintered Silver Nanoparticle Inks with Different Sizes during Furnace Sintering. Materials.

[68] Chen Z., Gengenbach U., Koker L., Huang L., Mach T.P., Reichert K.M., Thelen R., Ungerer M. (2024). Systematic Investigation of Novel, Controlled Low-Temperature Sintering Processes for Inkjet Printed Silver Nanoparticle Ink. Small.

[69] Späth A., Meyer M., Huthwelker T., Borca C.N., Meßlinger K., Bieber M., Barkova L.L., Fink R.H. (2021). X-Ray Microscopy Reveals the Outstanding Craftsmanship of Siberian Iron Age Textile Dyers. Sci. Rep..

[70] Bhushan B. (2008). Nanoscale Characterization of Human Hair and Hair Conditioners. Prog. Mater. Sci..

[71] Caballero-Díaz E., Pfeiffer C., Kastl L., Rivera-Gil P., Simonet B., Valcárcel M., Jiménez-Lamana J., Laborda F., Parak W.J. (2013). The Toxicity of Silver Nanoparticles Depends on Their Uptake by Cells and Thus on Their Surface Chemistry. Part. Part. Syst. Charact..

[72] Molleman B., Hiemstra T. (2015). Surface Structure of Silver Nanoparticles as a Model for Understanding the Oxidative Dissolution of Silver Ions. Langmuir.

[73] Hadrup N., Sharma A.K., Loeschner K. (2018). Toxicity of Silver Ions, Metallic Silver, and Silver Nanoparticle Materials after in Vivo Dermal and Mucosal Surface Exposure: A Review. Regul. Toxicol. Pharmacol..

[74] Samberg M.E., Oldenburg S.J., Monteiro-Riviere N.A. (2010). Evaluation of Silver Nanoparticle Toxicity in Skin in Vivo and Keratinocytes in Vitro. Environ. Health Perspect..

[75] Kazem Koohi M., Hejazy M., Asadi F., Asadian P. (2011). Assessment of Dermal Exposure and Histopathologic Changes of Different Sized Nano-Silver in Healthy Adult Rabbits. J. Phys. Conf. Ser..

[76] Danscher G., Jansons Locht L. (2010). In Vivo Liberation of Silver Ions from Metallic Silver Surfaces. Histochem. Cell Biol..

[77] Larese F.F., D’agostin F., Crosera M., Adami G., Renzi N., Bovenzi M., Maina G. (2009). Human Skin Penetration of Silver Nanoparticles through Intact and Damaged Skin. Toxicology.

[78] Lademann J., Richter H., Teichmann A., Otberg N., Blume-Peytavi U., Luengo J., Weiß B., Schaefer U.F., Lehr C.M., Wepf R. (2007). Nanoparticles—An Efficient Carrier for Drug Delivery into the Hair Follicles. Eur. J. Pharm. Biopharm..

