# Supplementary material for: Controlled Silver Nanoparticle Formation in Hair Fibers Dyed with Reseda luteola L.: A Study on Additive-Dependent Penetration and Aggregation
Source: Molecules. 2025 Aug 21;30(16):3446. doi: 10.3390/molecules30163446 (PMC12388297; doi:10.3390/molecules30163446)
Supplement: Supplementary file 1 [file molecules-30-03446-s001.zip › molecules-3769485-supplementary.pdf]

Controlled Silver Nanoparticle Formation in Hair Fibers Dyed with *Reseda luteola* L.: A Study on Additive-Dependent Penetration and Aggregration

Julia K. Hachmann[a], Charlotte Ruhmlieb[a], Volkmar Vill[a], Fabian Straske[a,b]

[a]University of Hamburg, Department of Chemistry, Hamburg, Germany

[b]Henkel AG & Co. KGaA, Hamburg, Germany

Corresponding Author: Fabian.Straske@henkel.com

Supporting Information

Table S1. Average nanoparticle size (mean ± SD) for strands adjusted to pH 3 using HCl unwashed and washed hair, as well as adjusted to pH 3 using CA. Each at 1, 4 and 8 µm measuring depth.

Entry	Localization inside hair[1]	mean particle size 0 washes, pH 3 HCl (n=67)	mean particle size 24 washes, pH 3 HCl (n=67)	mean particle size 0 washes, pH 3 CA (n=30)
1	1 µm	18.5±6.6 nm	30.5±12.4 nm	22.5±6.7 nm
2	4 µm	23.0±8.9 nm	40.0±17.4 nm	21.9±5.8 nm
3	8 µm	26.3±13.7 nm	50.2±24.1 nm	22.3±4.9 nm

[1] measuring depth starting from hair surface.

Table S2. Median values for strands adjusted to pH 3 using HCl unwashed and washed hair, as well as adjusted to pH 3 using CA. Each at 1, 4 and 8 µm measuring depth.

Entry	Localization inside hair[1]	median particle size 0 washes, pH 3 HCl (n=67)	median particle size 24 washes, pH 3 HCl (n=67)	median particle size 0 washes, pH 3 CA (n=30)
1	1 µm	16.2	33.2	20.4
2	4 µm	21.6	37.1	21.8
3	8 µm	22.3	46.9	20.4

[1] measuring depth starting from hair surface.

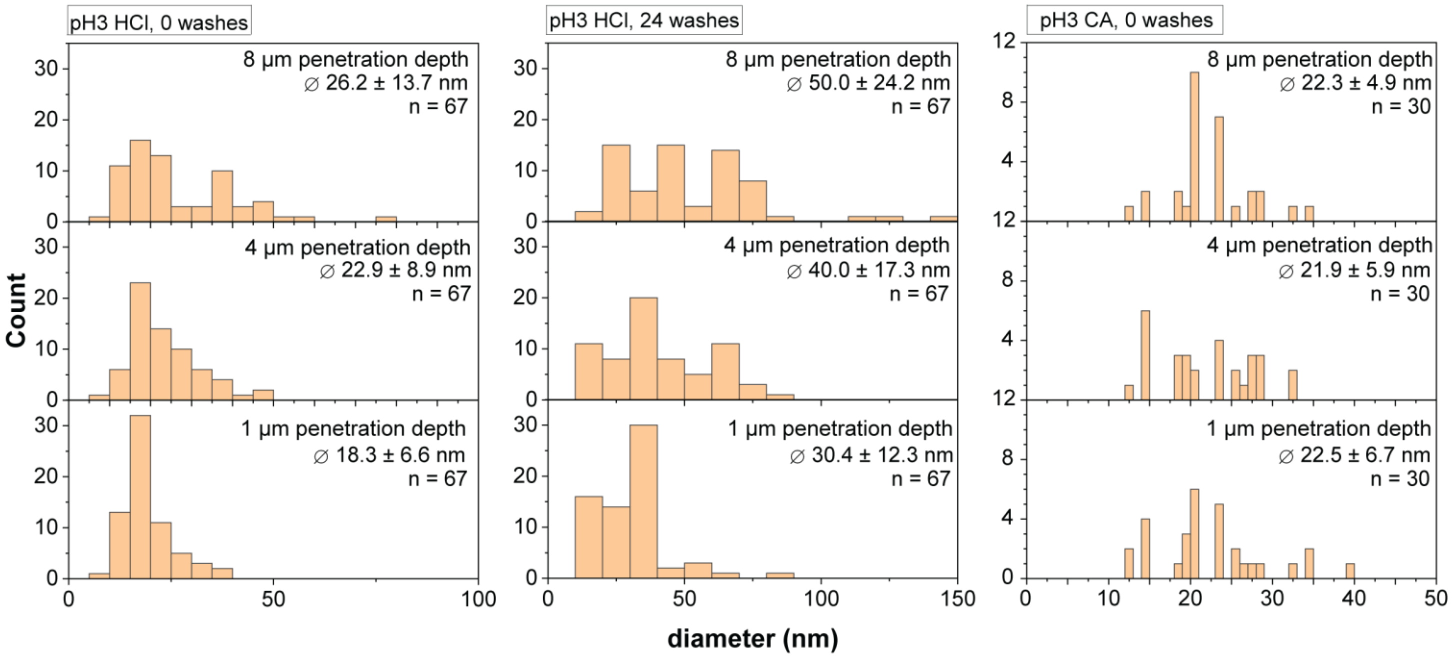

Figure S1. (A) Histograms depicting particle distribution, mean particle size (mean ± SD) and sample size (n).

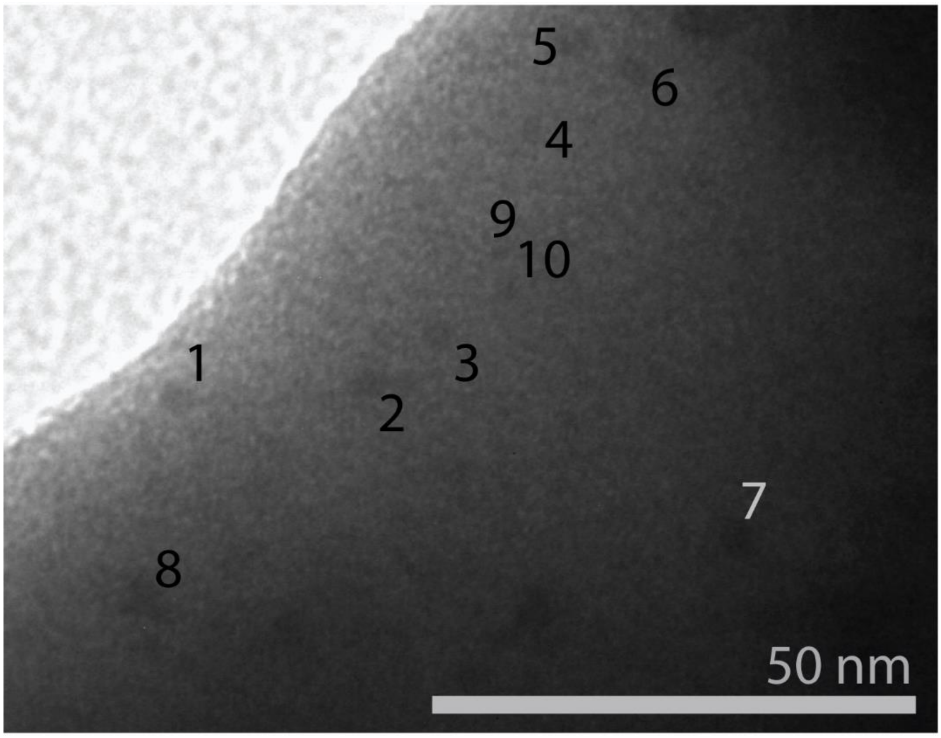

Figure S2. (A) Excerpt of TEM image of ground hair strand treated with 1% RE at pH3 adjusted with HCl (10%) and subsequent treatment with 1% $AgNO_3$. Numbers highlight the depicted particles.

Table S3. Particle diameters from Figure S2.

Entry	Particle diameter [nm]
1	3.94
2	3.83
3	2.98
4	1.42
5	3.85
6	2.41
7	3.01
8	3.31
9	1.91
10	1.28

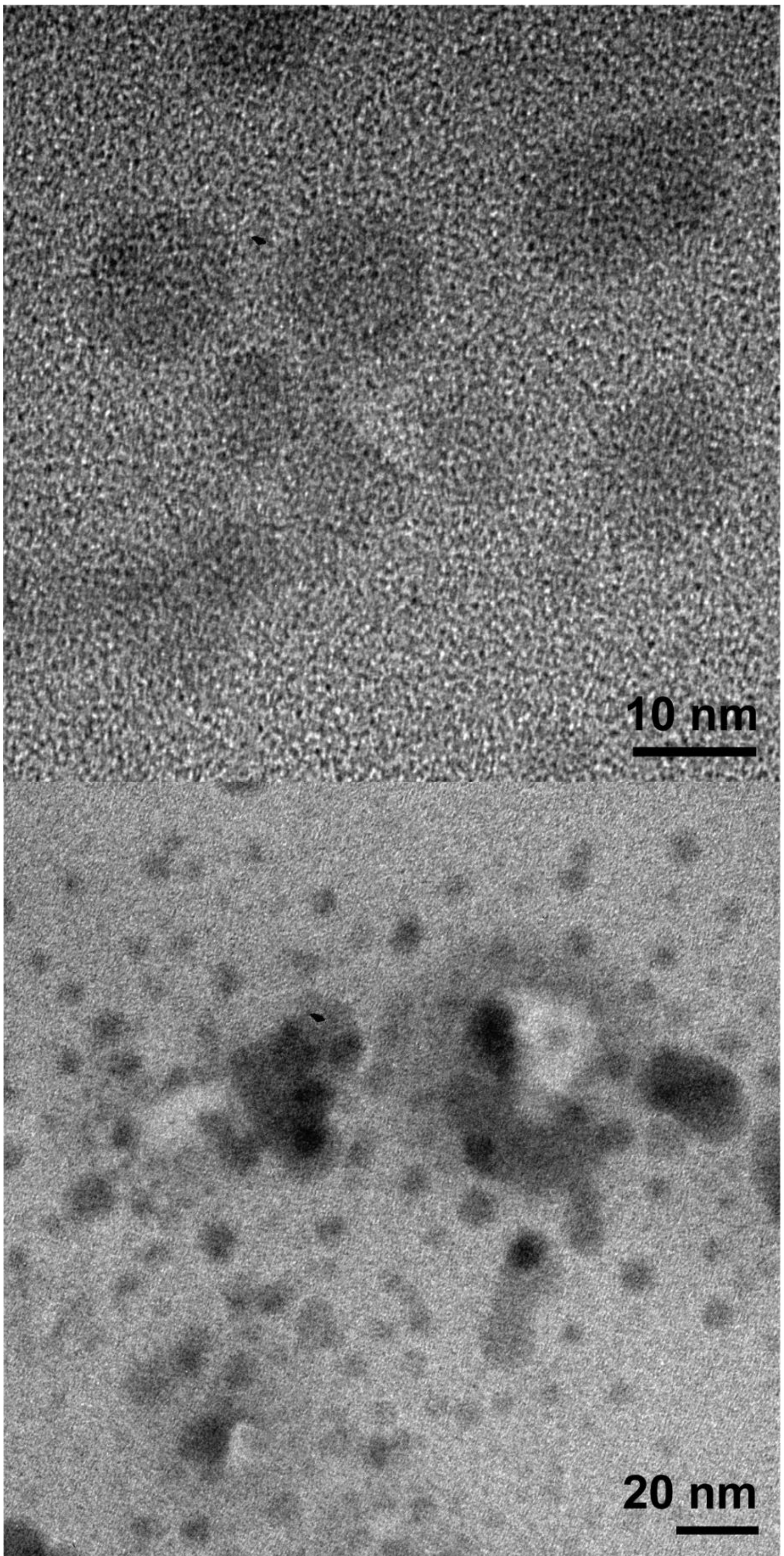

Figure S3: Excerpts of TEM images of microtome cut treated with 1% RE at pH 3 adjusted with 10% HCl and subsequent treatment with 1% $AgNO_3$ illustrating nanoparticles ≤ 10 nm.
